# Phase-selective graphene oxide membranes for advanced microfluidic flow control

**DOI:** 10.1038/micronano.2016.8

**Published:** 2016-04-11

**Authors:** Jennifer Gaughran, David Boyle, James Murphy, Robert Kelly, Jens Ducrée

**Affiliations:** 1 School of Physical Sciences, Dublin City University, Dublin 9, Ireland; 2 Biomedical Diagnostics Institute, Dublin City University, Dublin 9, Ireland

**Keywords:** centrifugal microfluidics, flow control, graphene oxide membranes, solvent selectivity

## Abstract

For the first time, we harness the unique phase-selectivity of chip-integrated graphene oxide (GO) membranes to significantly enhance flow control on centrifugal microfluidic platforms. In this paper, we present novel processes for the assembly of these GO membranes into polymeric microfluidic systems and demonstrate that multilayer GO membranes allow the passage of water while blocking pressurized air and organic solutions.

## Introduction

Flow control is of paramount importance for sample-to-answer automated Lab-on-a-Chip systems. Centrifugal microfluidics offers the benefit that the force field can be accurately controlled over several orders of magnitude by a spindle motor^1^. Such ‘Lab-on-a-Disc’ (LoaD) platforms have emerged as a highly useful tool in biomedical diagnostics because many of the necessary laboratory unit operations (LUOs) in bioanalysis, such as pumping^2^, plasma extraction^3^, mixing^4^, and metering^5^, can be fully automated and implemented. Liquid-handling steps can then be controlled by running a designated spin-frequency protocol to modulate interactions between on-disc liquids and microstructures^1^. The disc cartridge can also be designed to store all required reagents and then safely encapsulate potentially biohazardous samples^6^.

Due to the ubiquitous nature of the centrifugal field during rotation, flow control constitutes a particular challenge on integrated LoaD platforms. The coordination of sequential or parallelized LUOs (such as sample and reagent storage) requires high-performance flow control, such as passive and active valving.

Passive valves include capillary valves, hydrophobic valves, and siphon valves^7^. These types of valves are controlled primarily by the rotational frequency, surface properties, and channel design of the disc. Johnson *et al.*^8^ used capillary valves for the sequential handling of fluids. Siegrist *et al.*^9^ developed a more integrated system by implementing a robust serial siphoning method of fluid handling. This system was advanced further by integrating the serial siphoning system with a heterogeneous sandwich immunoassay and surface-confined supercritical angle fluorescence (SAF) detection of human immunoglobulin G (hIgG)^10^. Godino *et al.*^11^ integrated a paper siphon into a centrifugal LoaD platform, which introduced rotationally controlled bidirectional flow into the system. However, these valving schemes control only the liquid bulk, not its vapor phase.

Other valving mechanisms rely on the use of sacrificial materials for functional enhancement of microfluidic systems. These physical-barrier-based flow control elements are removed on demand by physical or chemical stimuli^12,13^. Wax valves, for instance, can be actuated by exposure to a heat source^13^. García-Cordero *et al.*^14^ integrated cyclo-olefin polymer (COP) and polyethylene terephthalate (PET) films, using laser printer lithography, as optofluidic valves that opened using a solid-state laser. Another example is hydrogels, which can act as valves when actuated by size change in the presence of water^15^. Hwang *et al.*^16^ incorporated an elastomeric membrane into a centrifugal microfluidic platform that could be controlled by the membrane thickness and rotational speed of the disc.

Gorkin *et al.*^12^ used a dissolvable film material that opens upon contact with the onboard liquid in conjunction with a pneumatic structure to develop a novel, essentially passive valving system on a centrifugal microfluidic platform. Nwankire *et al.*^17,18^ built on this system by integrating and automating multistep bioassay protocols actuated by the dissolvable-film-based centrifugo-pneumatic valving scheme. Kinahan *et al.* advanced this sacrificial technology to an event-triggered valving paradigm that enables the more dynamic control of fluids on a centrifugal microfluidic platform^19,20^. Dimov *et al.*^21^ combined a hydrophobic membrane and dissolvable film to improve on-disc solid-phase purification of nucleic acids. Although these sacrificial materials offer benefits for flow control, they often require some form of external stimulus for activatation; additionally, they are capable of acting on only one type of fluid (for example, hydrogels that activate only in the presence of water). The introduction of a single membrane that is capable of handling both aqueous and organic solutions would be of significant importance for the complex fluid handling requirements of numerous biological testing processes, particularly for nucleic acid testing.

Fluidic routing to direct flow to a selected output at a junction between a waste and an elution outlet is critical for centrifugally implemented automation, especially in the area of nucleic acid purification in which the sequence of fluids, some aqueous and some organic, must be passed through a single channel, but each of these fluids must be routed away from the final collection chamber. Kim *et al.*^22^ developed a flow switch by using a capillary valve upstream of an open chamber and unique three-dimensional (3D) junction geometry. A similar router, solely controlled by the rotationally actuated hydrodynamic Coriolis pseudo-force, was reported by Brenner *et al.*^23^. This virtual routing concept was further refined by Haeberle *et al.*, who successfully extracted DNA from calf thymus using silica beads by alternating the sense of rotation^24^. Automated extraction of human genomic DNA was demonstrated by Kloke *et al.*^25^, who implemented novel ball-pen pierceable seals to route the sample lysate through an integrated silica membrane in a Lab Tube platform. Although these routing systems are effective, they often required an external stimulus to direct the fluids. The combination of advanced routing solutions based on disc-integrated functional materials could enable more dynamic fluid control.

In this work, we harness graphene oxide (GO)-based membranes for advanced flow control on LoaD platform. As its ground-breaking discovery in 2004 (Ref. 26), a wide spectrum of fascinating characteristics of graphene and its compounds, such as the GO considered here, have been extensively investigated by the scientific community. In recent years, microfluidic systems have been used to characterize the distinctive properties of graphene, such as its electrochemical responses^27^. Ang *et al.*^28^ used a graphene transistor array in a microfluidic chip for the detection of malaria-infected red blood cells. Lo *et al.*^29^ included glycidyl methacrylate-functionalized graphene oxide within a hydrogel, which showed a significant increase in size change when exposed to infrared radiation. However, although graphene oxide is under investigation by a large scientific community, its often unique properties have not yet been used for microfluidic flow control. This lack of use for microfluidic flow control is presumably because GO does not bind well to polymer surfaces. To address this issue, we first developed a scheme for the integration of GO membranes in common, polymeric microfluidic devices.

**Figure 1:**
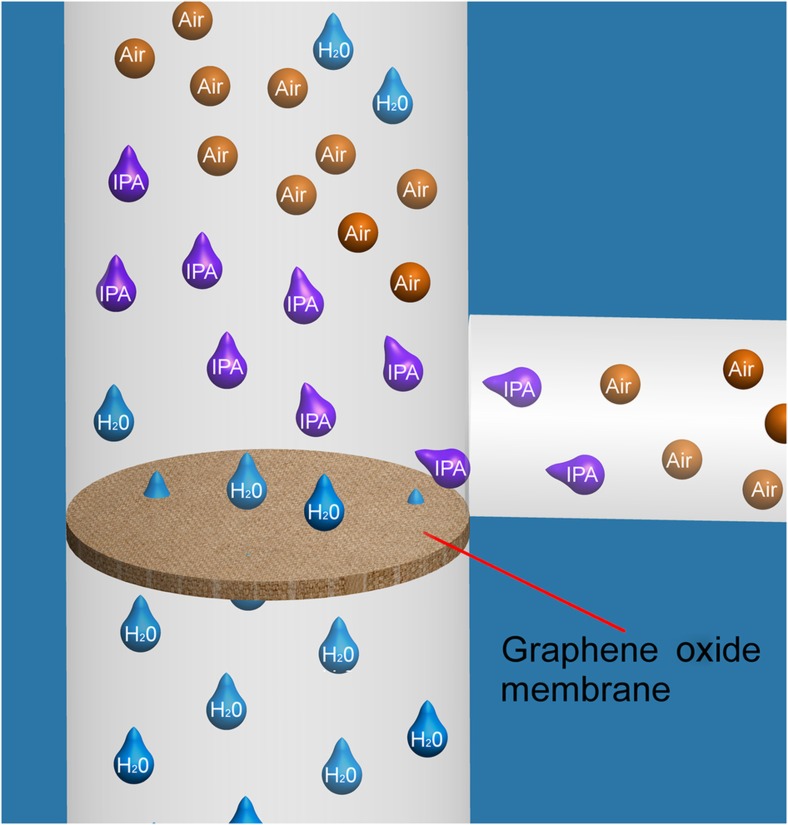
Illustration of the unique properties of the graphene oxide membrane. The GO membrane is entirely permeable to water (blue) but is completely resistant to organic solutions (IPA and EtOH; purple) and air (orange).

The properties of such integrated GO membranes are anticipated to enable a multitude of applications and advanced flow control. We demonstrate two such properties of the GO membranes: their solvent selectivity and air impermeability (Figure 1). Beyond a specific burst pressure, GO membranes allow water to pass through at very low flow resistance while completely blocking the organic solutions, isopropyl alcohol (IPA) and EtOH, or air, even at high pressure heads. Nair *et al.* postulated that the spacing between the GO flakes in multilayers act as nanocapillaries that allow the low-friction flow of a monolayer of water; the stacked membrane is analogous to a monolayer of water (≈5 Ǻ)^30^.

We employed these unique solvent- and phase-selective features of GO to show, for the first time, graphene-oxide-enabled advanced centrifugal flow control.

## Materials and methods

**Figure 2:**
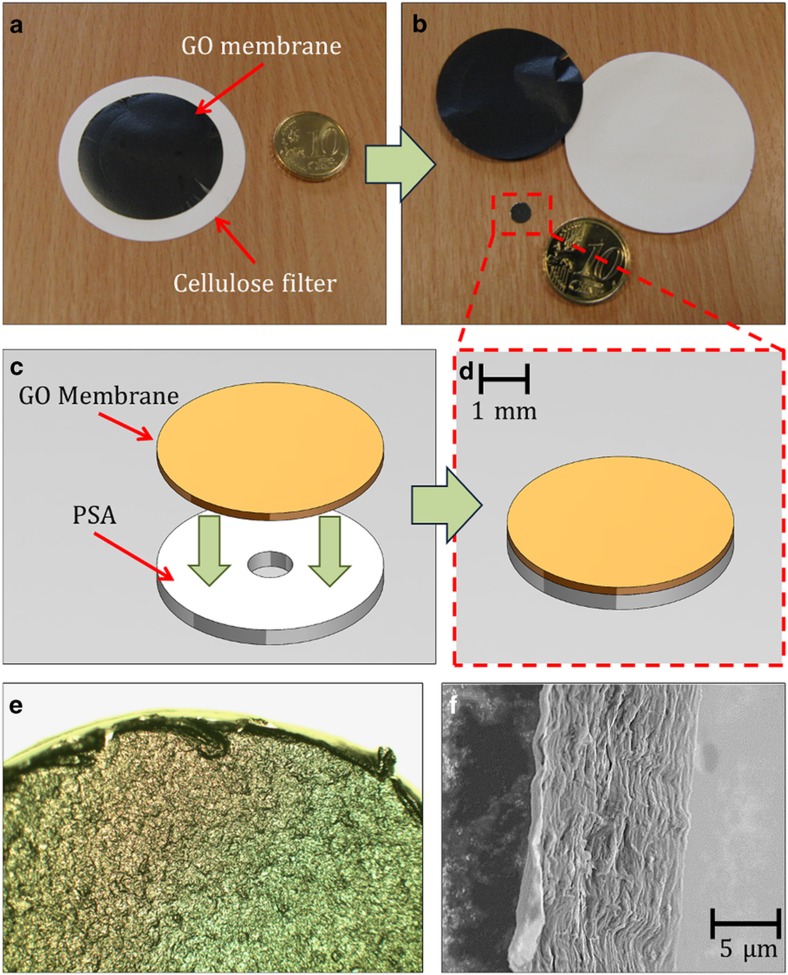
GO tab assembly and characterization. (**a**) Image of the GO membrane and the cellulose filter after vacuum filtration. (**b**) Free-standing membrane removed from filter after drying and a GO tab after fabrication. (**c** and **d**) Assembly of GO tab. The GO membrane is adhered to double-sided PSA with a 1-mm through-hole cut out. (**e**) Microscope image of the GO tab edge showing uniform distribution of flakes and the acceptably clean cut from the knife cutter. (**f**) SEM image of the GO membrane showing a multilayer stacked structure.

The membranes were synthesized from a suspension of GO flakes in water (Bluestone, Manchester, UK). The suspension was first compressed by vacuum filtration through a 0.45-μm pore size cellulose filter (Millipore, Cork, Ireland). As they filled the pores of the cellulose, the flakes formed a multilayer structure across the entire filter surface. This process eventually created a free-standing, 35-mm diameter GO-filter hybrid, whose diameter was defined by the vacuum filtration apparatus. The thickness of the membranes was governed by the volume and concentration of the GO flakes in suspension. The process time ranged from 4 to 8 h, depending on the volume being filtered (Figure 2a).

After completion, the membranes were removed from the vacuum filtration setup and left to dry for ~4 h. During this time, the membranes remained in contact with the cellulose filters. The completion of this drying step was crucial because prior handling of the membranes could lead to tearing. The membrane was then peeled from the cellulose filters and handled with ease (Figure 2b).

To incorporate the GO into a microfluidic device, a method for attaching the membrane to the polymer surface had to be devised. Using a method adopted from Gorkin *et al.*, the membrane was adhered to double-sided pressure-sensitive adhesive (PSA), which featured a small, 1-mm through hole. The contours and size of the combined GO and PSA ‘tab’ were then flexibly defined using a precision knife cutter (Graphtec, Craft Robo Pro, Wrexham, UK). Next, the GO tab was integrated into a microfluidic system using ‘sticky’ PSA backing (Figures 2c and d).

Figure 2f shows an SEM image of a cross section of a GO membrane, which clearly displays the multilayer structure. This stacked membrane possesses a thickness of ~10 μm. From this image, the even distribution of the flakes can be observed. Also shown in this figure is the relative clean cut of the tab edge, achieved using a precision knife cutter (Graphtec, Craft Robo Pro, Wrexham, UK).

## Results

Two distinct properties of the GO were investigated: its air impermeability and solvent selectivity. These properties were tested over a given pressure range on two centrifugal microfluidic discs.

### Air Impermeability

**Figure 3:**
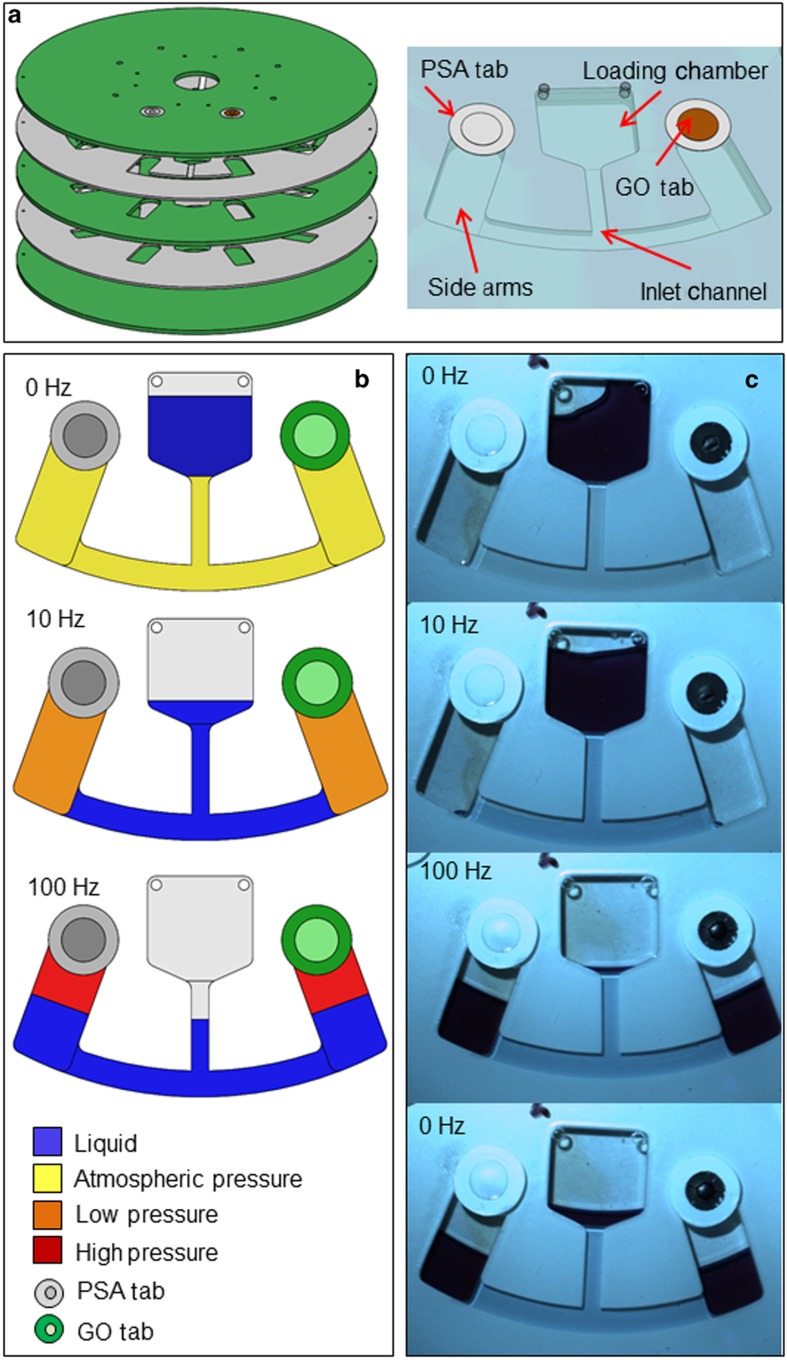
Measurement of GO impermeability to air. (**a**) Exploded view of disc design with PMMA (green) and PSA (gray) layers and design structure showing the functional parts. The left and right arms are sealed by PSA and GO tabs, respectively. (**b**) Operation under various air pressures. (**c**) Image sequence showing that even at high-speed rotation at 100 Hz, hydrostatic equilibrium is maintained to demonstrate the impermeability of the GO membrane to air (and the integrity of the membrane and its seal with the disc substrate).

Figure 3 shows the design for testing the impermeability of the GO tabs to air. This disc consisted of three layers of PMMA and two layers of PSA (Figure 3a). The discs contained four identical structures, each of which featured a loading chamber and two symmetric side arms that were connected via a common inlet channel. Both side arms exhibited a hole at the top, which had been sealed by either a PSA or GO tab (Figure 3a). For the sake of clarity in these tests, colored food–dye in water was used, although the liquid would not make direct contact with the tabs.

A 180-μl volume was placed in the loading chamber. This liquid trapped and hence enclosed the air inside the inlet channel and side arms. With the increasing rotationally induced centrifugal field, liquid extends down the inlet channel to compress the air in the side arms. The liquid reached the interface between the channel and side arm at a rotational frequency of 10 Hz. As the disc was spun faster up to a rotational frequency of 100 Hz, the liquid reached the side arms and continued to rise (Figure 3b). Figure 3c shows that there was no discernible difference of the liquid levels in both side arms between the upper spin frequency and when the spin frequency was once again reduced to 0 Hz, thereby proving the air impermeability and pressure tightness of the GO tab.

Another interesting feature is the relatively high rotationally induced pressures

(1)Δpω=ρΔrr¯ω2

where Δ*p*_*ω*_ is the burst pressure in pascals, *ρ* is the density of the liquid, Δ*r* is the length of the liquid plug, $\bar{r}$ is the distance of the tab from the centre of rotation, and *ω* is the angular frequency that the tabs are able to withstand.

Upon loading, the liquid sealed the air inside the channel and arms essentially under atmospheric pressure. As the spin frequency was increased to 10 Hz, the air pocket sealed by the GO tab was compressed to ~25 mbar (obtained using Boyle’s law). Finally, at 100 Hz, there was a significant increase in pressure of ~750 mbar. This result verifies the sufficient mechanical strength of the integrated GO membranes. This result can be observed in Figure 3c, in which the GO tab bends without disruption.

### Solvent selectivity

**Figure 4:**
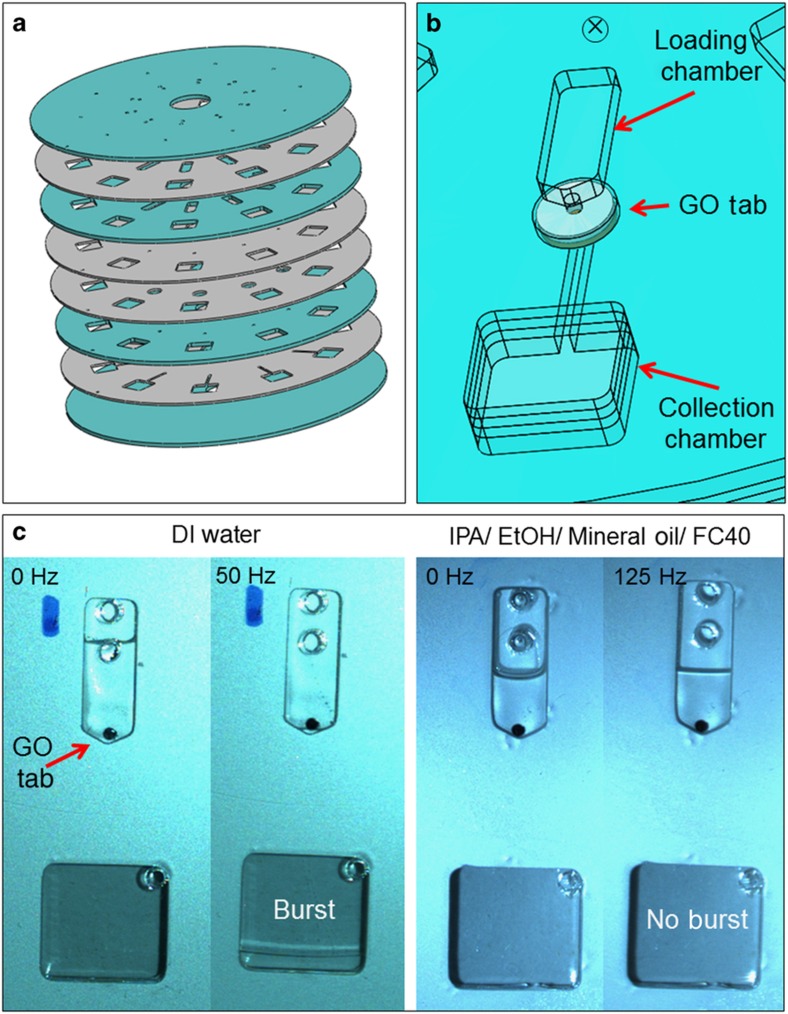
Design for testing the solvent selectivity of the GO membrane. (**a**) Multilayer disc structure, with PMMA (green) and PSA (gray) layers. (**b**) Three-dimensional architecture connecting the loading chamber to the collection chamber via a channel and through hole, sealed by a GO tab. (**c**) Solvent selectivity of GO. Deionized (DI) water passes through at 50 Hz and the membrane is impermeable to the organic solvents, IPA and EtOH, and the oil solutions, FC40 and mineral oil, even at the much higher spin frequency of 125 Hz. Both the organic and oil solutions are clear liquids, therefore, a representation of their flow is shown here.

Figure 4 shows the disc used to test the solvent selectivity of the GO. The disc was made up of four layers of Poly(methyl methacrylate) (PMMA) (green) and four layers of PSA (gray). It contained 10 identical structures. Each structure consisted of a 3D architecture containing a loading chamber with a 1-mm hole at the base of the chamber. This vertical through-hole is connected to a channel on the bottom layer of the disc. The hole was sealed by one of the GO tabs. The channel is then connected to a collection chamber (Figure 4b).

To test the solvent-selective properties of the GO, five liquids were added to the chambers: deionized (DI) water, 2-propanol (IPA), ethanol (EtOH), Fluorinert FC40 (Sigma-Aldrich, Arklow, Ireland) and mineral oil. A 50-μl volume was added to the loading chamber. Under centrifugation, the liquid generated a hydrostatic pressure head acting on the GO tab. The rotational frequency was increased at regular intervals until the membrane yielded. In the case of DI water, the plug passed through the GO at ~50 Hz (Figure 4c). Notably, once the ‘burst’ pressure was exceeded, the water penetrated with very low flow resistance. By contrast, the organic solutions IPA and EtOH and the oil solutions FC40 and mineral oil, were fully retained in the loading chamber, even at the highest spin frequency of 125 Hz that we could implement on our test stand.

This simple test demonstrates the complete impermeability of the GO membrane to the organic solutions IPA and EtOH and the oil solutions FC40 and mineral oil, while offering very little flow resistance to water above a certain pressure threshold.

### Characterization of GO tabs

Using the structure shown in Figure 4, the capabilities of the solvent-selective nature of the GO tabs was investigated. First, because the membranes had been shown to be permeable to water but completely impermeable to IPA, water and IPA mixtures were tested. Solutions with varying water concentration in IPA were flowed through the GO tabs, up to the water concentration at which a burst could no longer be achieved. The tab used in these experiments was ~10-μm thick.

**Figure 5:**
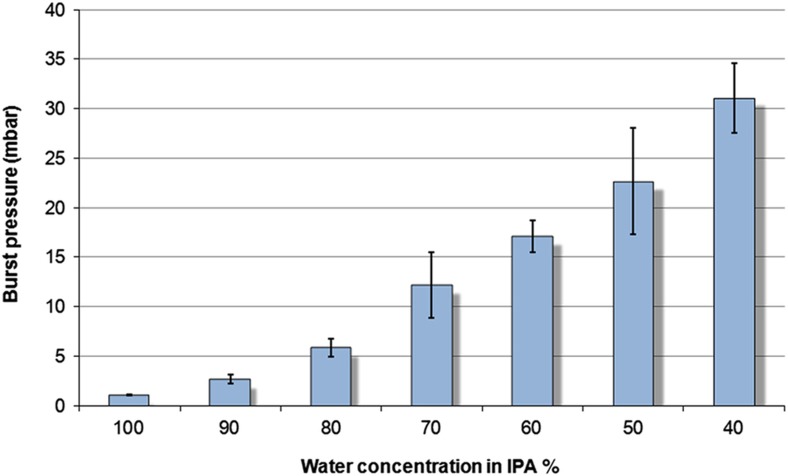
Change in burst pressure required for the passage of fluid with varying water concentrations in IPA. The data show an inverse relationship between the water concentration in the solution and burst pressure require for flow. Data points are mean±1 standard deviation, *n*=5.

The burst pressure, Equation (1), required for flow increases with decreasing water content (Figure 5). It was also determined that any solution with a concentration less than 40% water was incapable of passing through the GO tab, even at the highest spin frequency we could realize for safety reasons.

**Figure 6:**
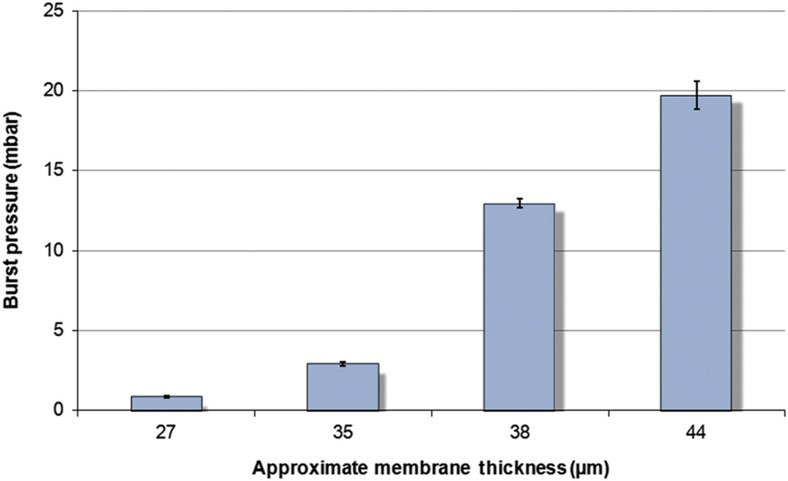
Change in burst pressure required for the passage of fluid with varying membrane thicknesses. The data show an inverse relationship between the thickness of the membrane and burst pressure required for flow. Data points are mean±1 standard deviation, *n*=5.

The second experiment revealed the correlation between the thickness of the GO membrane and the burst pressure for water. The variation in the tab thickness was achieved by filtering different concentrations of GO flakes (Figure 2). In this case, four different membrane thicknesses were used, approximately 27, 35, 38, and 44 μm. The thickness of the GO membrane was then measured using a 3D microscope (Keyence, VHX-5000 series, Digital Microscope, Itasca, IL, USA). For this experiment, a 100% DI water solution was used, and the burst frequency required for flow was once again recorded and converted to burst pressure using Equation (1). As seen in Figure 6, the burst pressure increases with increasing thickness of the membrane.

These two experiments demonstrate the dynamic range of capabilities of the GO tabs. By varying their thickness, the tabs can be tailored to work for a multitude of designs within a centrifugal system.

## Conclusions

We have shown a new method for the integration of a GO tab within a polymeric microfluidic structure. Using centrifugal flow control, we have furthermore investigated various unique features of GO, notably its solvent selectivity and air impermeability. We envision the engineering of a solvent-selective burst valve based on this surprising, selective permeability of GO membranes. The tabs also offer avenues for enhanced centrifugal flow control towards comprehensive assay automation on the Lab-on-a-Disc platform.
